# 

**Published:** 1908-01

**Authors:** 


					﻿Sixty-third Year.
Vol. LXIII.	JANUARY. 1908.	No. 6
BUFFALO
MEDICAL JOURNAL
Established 1845 by AUSTIN FLINjT 4- IdR P \ |
EDITOR:	I DEC. 3Q
WILLIAM WARREN POTTER, IM. D.
• * V y	r	I •	.
~^ar" -	ASSISTANT EDITORS :	--— 
WILLIAM C. KRAUSS, M. D.	NELSON W. WILSON, M. D.
ASSOCIATE EDITORS:
JAMES WRIGHT PUTNAM, M. D. ERNEST WENDE, M. D.
JOHN PARMENTER, M. D.	JOHN A. MILLER, Ph. D.
MAUD J. FRYE, M. D.
				

